# An Ionic-Polymer-Metallic Composite Actuator for Reconfigurable Antennas in Mobile Devices

**DOI:** 10.3390/s140100834

**Published:** 2014-01-06

**Authors:** Yi-Chen Lin, Chung-Yi Yu, Chung-Min Li, Chin-Heng Liu, Jiun-Peng Chen, Tah-Hsiung Chu, Guo-Dung John Su

**Affiliations:** 1 Graduate Institute of Photonics and Optoelectronics, National Taiwan University, No. 1, Sec. 4, Roosevelt Rd., Taipei 106, Taiwan; E-Mails: victorlin1020@gmail.com (Y.-C.L.); r01941078@ntu.edu.tw (C.-Y.Y.); r01941089@ntu.edu.tw (C.-M.L.); r99941044@ntu.edu.tw (C.-H.L.); 2 Graduate Institute of Communication Engineering, National Taiwan University, No. 1, Sec. 4, Roosevelt Rd., Taipei 106, Taiwan; E-Mails: jpchen.home@msa.hinet.net (J.-P.C.); thc@ntu.edu.tw (T.-H.C.)

**Keywords:** reconfigurable antenna, RF switch, electro-active polymer actuator

## Abstract

In this paper, a new application of an electro-active-polymer for a radio frequency (RF) switch is presented. We used an ionic polymer metallic composite (IPMC) switch to change the operating frequency of an inverted-F antenna. This switch is light in weight, small in volume, and low in cost. In addition, the IPMC is suitable for mobile devices because of its driving voltage of 3 volts and thickness of 200 μm. The IPMC acts as a normally-on switch to control the operating frequency of a reconfigurable antenna in mobile phones. We experimentally demonstrated by network analysis that the IPMC switch could shift its operating frequency from 1.1 to 2.1 GHz, with return losses of than −10 dB at both frequencies. To minimize electrolysis and maximize the operation time in air, propylene carbonate electrolyte with lithium perchlorate (LiClO_4_) was applied inside the IPMC. The results showed that when the IPMC was actuated over three months at 3.5 V, the tip displacement fell by less than 10%. Therefore, an IPMC actuator is a promising choice for application to a reconfigurable antenna.

## 1. Introduction

Polymers have attractive advantages; they are soft, flexible, and lightweight. Some polymers can even change in size or shape under electrical stimulation. These are called electro-active polymers (EAPs). EAPs can provide large deformation with low driving voltage and produce force as actuators. Compared to conventional methods such as switches and mechanical actuators, EAPs have some attractive advantages like small electrical energy consumption, light weight and compliant properties, biocompatibility, ability to operate in air and aquatic media, insensitivity to magnetic fields and simple fabrication processes, making them suitable for actuators and sensors [1]. Based on the driving mechanism, EAPs can be classified into two major types: electronic EAPs and ionic EAPs [2]. Electronic EAPs can generate greater force than ionic EAPs and hold induced displacement under an electric field, but they require a high driving voltage. The deformation mechanism of electronic EAPs is the Coulomb force, including dielectric, electrostatic, piezoelectric and ferroelectric forces. Ionic EAPs generate deformation by movement or diffusion of ions between two electrodes. They can induce bending displacement at relatively low voltage, but at such a voltage, it is hard to make them maintain the same position. Popular ionic EAPs include ionic polymer gels, ionic polymer-metal composites, conductive polymers, and carbon nanotubes. Generally speaking, ionic EAPs can deform greatly when they are stimulated by electric signals.

An ionic polymer metallic composite (IPMC) consists of an ion-exchange polymer membrane, two coated electrodes, and mobile cations. An IPMC can bend gradually with increasing voltage. When the applied voltage is off, the IPMC is flat. When the voltage is on, the hydrated positive ions (Na^+^, Li^+^, H^+^) carry water and move toward the anode. This mechanism of ionic motion causes the IPMC to bend [3]. An IPMC can be made into an actuator, and the actuator can be used in several applications, such as biomechanics, grabbers, and robotics.

The antenna design of portable devices is important. Antennas receive different signals according to their lengths in order to ensure good communication quality. Long antennas are used for low frequency bands. A cell phone is usually designed to receive many bands, such as GSM (Global System for Mobile Communications) 900, GSM 1800, Bluetooth, and local wireless. However, cell phones must also be small to satisfy the consumer. Thus, it is challenging for designers to integrate various antennas. We propose a concept to connect short antennas into one long antenna, or a reconfigurable antenna. A reconfigurable antenna has controllable parameters, such as the radiation pattern or operating frequency. Using this type of antenna allows effective use of the inner space of the cell phone. It is well known that changing the length of an antenna can shift the operating frequency. In order to design an antenna that can change size, RF switches have been introduced. An RF switch is used to change the radiation pattern or operating frequency [4–8]. Generally speaking, RF switches use PIN (positive-intrinsic-negative) diodes and GaAs transistors to control the signal, but those switches need relatively high power and provide low isolation. RF MEMS (micro-electro-mechanical systems) switches have also been proposed [9,10]. RF MEMS switches have low power consumption, low insertion loss, and high isolation, and they play an important role in RF devices. Although RF MEMS switches can shift the frequency of the antenna, the required driving voltage, at least nine volts [11], is considered high for portable devices. A common portable device can offer about 3 volts for switching, so the high driving voltage of MEMS switches is not ideal for portable devices. In order to overcome this problem, this paper introduces an IPMC actuator used as a switch. The low driving voltage and large tip displacement of IPMC actuators result in high isolation.

**Figure 1.**
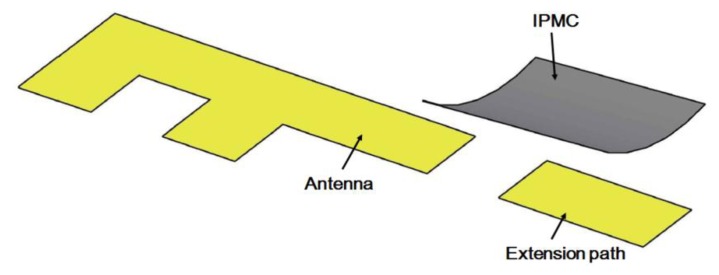
Schematic representation of one IPMC switch moved to control the operating band of an antenna.

The operating frequency of an antenna is dependent on its physical length. To design a switch that can change the band of an antenna, we propose a bridge structure. The concept is to use an IPMC to connect two antenna paths, which can extend the physical length of the antenna and change the operating frequency between the high and low bands. This device consists of one antenna, an extension path, and a movable bridge controlled by the IPMC actuator, as shown in Figure 1. In this paper, we first show how to make IPMC switches. Then, we discuss the design concept of a reconfigurable antenna device using an IPMC switch. The simulation and experimental results show that a small driving voltage can be used to shift the operating frequency of the IPMC switch. Regarding the reliability, we know that the IPMC must work in wet conditions because it needs water molecules to bend. When an IPMC is used in mobile devices, however, it is actuated by voltage and operates in the air, where electrolysis and evaporation could occur. To solve these problems, a water solution was replaced in this study with propylene carbonate. We demonstrate herein that this is a promising solution to designing a reconfigurable antenna. Section 2 and Section 4 are related to characteristics and reliability of IPMC. While the antenna design and simulations are given Section 3. Finally, the paper is concluded in Section 5.

## 2. Ionic Polymer Metallic Composite Switch

### 2.1. Fabrication Process

**Figure 2.**
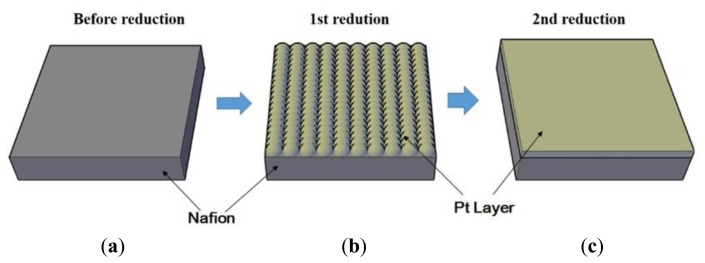
IPMC fabrication process. (**a**) Step 1: surface treatment; (**b**) Step 2: first plating and (**c**) Step 3: second plating.

To fabricate an IPMC actuator, chemical decomposition, also known as electroless-plating, is required [12]. It is a method that utilizes reduction from a metal complex solution. This process has three major steps: surface treatment, first reduction, and second reduction. We used Nafion™-117 as an ion-exchange membrane and a platinum layer as electrodes. The fabrication flow is illustrated in Figure 2.

Step 1: The first step of the IPMC fabrication was to roughen the surface of the ion-exchange membrane by sand blasting to increase the interfacial area density between the polymer membrane and metal composites. To remove any particles and impurities, we used ultrasonic washing and chemical treatment with boiled hydrochloric acid (low concentration) and boiled de-ionized water (DI water), respectively. Ion-adsorption was accomplished by immersing the membrane in platinum complex solution [Pt(NH_3_)_4_Cl_2_], and ammonium hydroxide solution (NH_4_OH) was introduced to neutralize the solution before the membrane was left to stand at room temperature for at least one night.
Step 2: Before the first plating, we immersed the membrane in DI water at 40 °C and added 2 mL of the aqueous sodium borohydride solution (NaBH_4_, 5 wt%) and 20 mL of the NaBH_4_. We added NaBH_4_ every 30 min while gradually raising the temperature to 60 °C. As a result, a dark layer of Pt particles was deposited on the surface of the membrane, as shown in Figure 2b. Then the membrane was removed and rinsed with water, after which it was immersed in dilute HCl (0.1 M) for at least eight hours. The primary reaction for this process is:
  (1) $${\text{NaBH}}_{4}+4{\left[{\text{pt}(\text{NH}}_{3})\right]}^{2+}+8{\text{OH}}^{-}\to 4{\text{Pt}+16\text{NH}}_{3}+{\text{NaBO}}_{2}+6{\text{H}}_{2}\text{O}$$
Step 3: In the first plating, only a very thin platinum (Pt) layer was deposited. Therefore, the next step was to improve the electroplating process by plating a thicker layer of platinum by secondary reduction. Aqueous solution of the complex [Pt(NH_3_)_4_]Cl_2_ was prepared, and 0.6 mL of the 25% ammonium hydroxide solution was added. Also, a 5 wt% aqueous solution of hydroxylamine hydrochloride (H_2_NOH·HCl) and 20% solution of hydrazine (H_2_NNH_2_·H_2_O) were prepared. The membrane was placed into the Pt solution at 40 °C, and 3 mL of hydroxylamine hydrochloride solution and 1.5 mL of the hydrazine solution were added every 30 min for 4 h as the temperature was gradually increased to 60 °C. During this process, the surface electrodes of metallic particles grew smoothly on both surfaces of the IPMC.
Step 4: After the IPMC was rinsed with water and boiled in dilute HCl (0.1 M), it was stored in sodium hydroxide solution (0.1 M) for 3 days. Na^+^ in the composite can be exchanged for any cation by immersing the IPMC in a solution of another cation, such as LiOH, which can be used to replace the Na^+^ with lithium cations.
Step 5: Propylene carbonate (99.5% w/w) 25 mL was prepared as solvent and 0.6 M lithium perchlorate (LiClO_4_) was added. Then the IPMC was immersed in the propylene carbonate solution for 3 days.

### 2.2. Morphology Measurement

**Figure 3.**
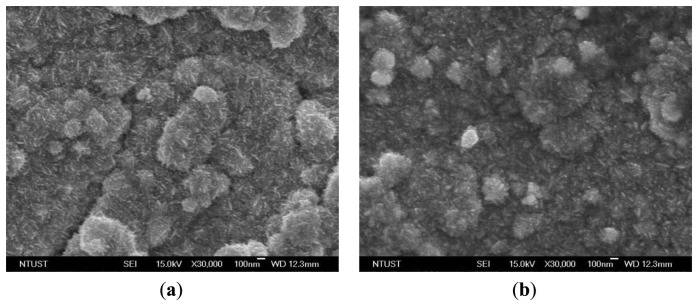
Top-view SEM micrographs of surface electrodes after (**a**) initial plating (magnification 30,000×) and (**b**) second plating (magnification 30,000×).

With the naked eye, it was not easy to recognize the differences between the electrode surfaces after the first plating and the second plating. Both electrodes were silver in color. Figure 3 shows top-view SEM micrographs (JSM-7600F, JEOL, Tokyo, Japan) of the IPMC surface electrodes after the initial plating and the second plating. The magnification is 30,000 times at 15 kV. In Figure 3a, it can be seen that the platinum particles were lumps and the cracks were deep. After the second reduction step, however, the platinum particles were small and the cracks were fine, as shown in Figure 3b. The samples were cut into pieces of 10 mm × 5 mm and the surface resistance was measured on the diagonal. The surface resistances with the initial plating and with the second plating were about 22.6 and 6.4 Ω, respectively. These results showed that after the second chemical plating, the surface electrodes were smoother and surface resistance was reduced. These conditions reduce evaporation of the solution in the ion exchange membrane.

### 2.3. Tip Displacement of IPMC

**Figure 4.**
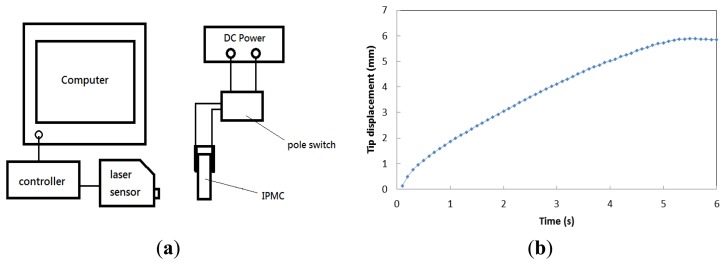
(**a**) Schematic diagram of tip displacement measured by laser sensor; (**b**) Diagram of tip displacement of IPMC switch *versus* response time at 3 V.

Tip displacement was measured with a model number LK-G80 laser displacement sensor (Keyence, Itasca, IL, USA). The setup is illustrated in Figure 4a. The IPMC was actuated by a voltage source, and the laser sensor detected the position of the end of the IPMC during the operating period. Via a controller, the position data was sent to a computer for recording and analysis. Figure 4b shows the tip displacement of the IPMC switch *versus* time. The applied voltage was 3 V. We found that tip displacement was close to 2 mm at 1 s and reached the maximum value of about 6 mm in 6 s; the corresponding velocity was 1 mm/s. This result ensured that the IPMC switch would have two antenna paths of over 1 mm for changing of the operating frequency, as discussed in the next section.

## 3. Reconfigurable Inverted-F Antenna

### 3.1. Design Concept

**Figure 5.**
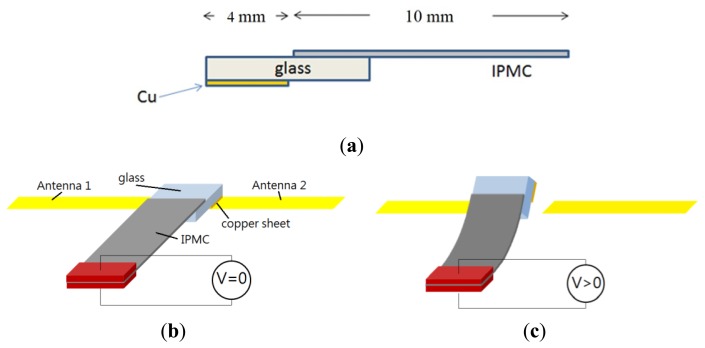
The illustration of normally-on IPMC switch design: (**a**) cross-section; (**b**) the on state (*V* = 0) and (**c**) the off state (*V* > 0).

In order to prevent electric current from being drawn directly into the antenna, the IPMC could not be used directly as a switch. Indeed, the metal electrodes on the IPMC could affect antenna performance due to the metal shielding effect. We used an electrical-isolation material, a thin piece of glass, to separate the IPMC and copper sheet, as shown in Figure 5a. The thickness of the glass with the copper sheet was 1.145 mm, considering the weight of glass and cooper sheet, IPMC should be large enough to offer needed force to handle the structure. At the same time, the space was limited in a mobile phone. We chose a 7 mm × 10 mm IPMC to make a reconfigurable antenna. If we decreased the size of the IPMC, the force might be too small to contact the antenna properly.

We know that the IPMC will bend toward the positive electrode, so it is suitable for use as either a normally-on switch or a normally-off switch. In our proposed configuration, the IPMC-bridge was a normally-on switching device. When there was no applied voltage, the IPMC switch touched the antenna and was pressed by a load to avoid backward relaxation issue. The extended path was in a conducting state, shown in Figure 5b as on-state. When the voltage was applied, the IPMC switch bent away from the device and the entire system was an open circuit, shown in Figure 5c as off-state. Due to this feature, we were able to design an extra extension path for the antenna and use this IPMC switch to connect it. The backward relaxation appeared in off-state and it didn't affect our control to change frequency because the distance between the actuated IPMC and the antenna was more than 1 mm.

### 3.2. Simulation Results

**Figure 6.**
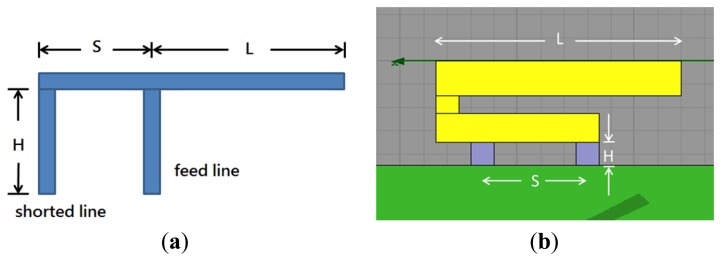
(**a**) Structure of inverted-F antenna; and (**b**) Simulation layout with substrate board.

The antenna simulations were performed with the high frequency structure simulator (HFSS) developed by Ansoft^®^ (Canonsburg, PA, USA). We used a popular antenna style, the inverted-F antenna, as our major design. The inverted-F antenna is widely used in wireless communication because of its low profile, low weight, and easy fabrication. We can pre-calculate the operating frequency by empirical formula. The three main parameters that decide the operating frequency are illustrated in Figure 6a. We can vary the combination of the three parameters to select the desired operating frequency and adjust the input impedance of the antenna to about 50 Ω of pure resistance. This removes the need for another impedance-matching circuit to match the microwave transmission line. To install the inverted-F antenna in a mobile phone model, we folded the upper arm of the antenna and assumed the parameter *L* to be the length of the new upper arm, as in Figure 6b. The structural parameter *S* in our design was 9 mm, *L* was 21 mm, and *H* was 2 mm. *L* + *H* were roughly a quarter of the wavelength, which provided an operating frequency of 2.1 GHz and characteristic impedance near 50 Ω. The material of the antenna, feed line, ground line, and ground plane were copper with very high electrical conductivity, and the dielectric substrate was FR-4 epoxy, which is often used in printed circuit boards.

**Figure 7.**
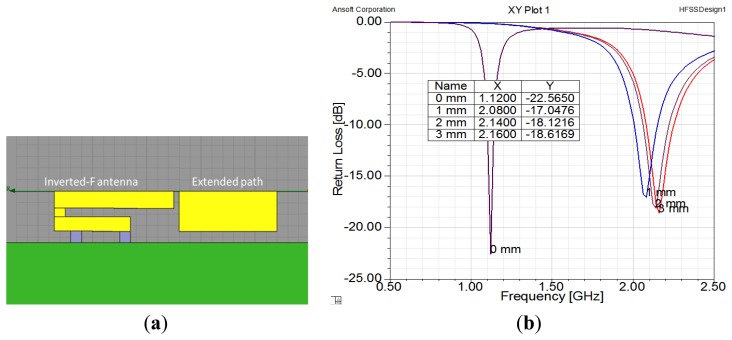
(**a**) Design of extended path; (**b**) Optimization of gap between two antenna strips. The horizontal axis represents frequency and the vertical axis represents return loss.

The purpose of our design concept, which is illustrated in Figure 1, is to extend the length of an antenna by using a switch. Therefore, it is necessary to set an extended path for connection. In order to design the extended path of an antenna, we used simulations to check the gap size, as shown in Figure 7. When there was no gap, the extended path was connected with the inverted-F antenna and the antenna was 40 mm long. The operating frequency was 1.1 GHz. When the gap was greater than 1 mm, the antenna was shorter and the operating frequency was around 2.1 GHz. Thus, we set the gap at 1 mm. Please note that we chose these frequencies for demonstration purposes; operating frequencies of 900 MHz and 1800 MHz can be achieved by adjusting the length of the antenna.

**Figure 8.**
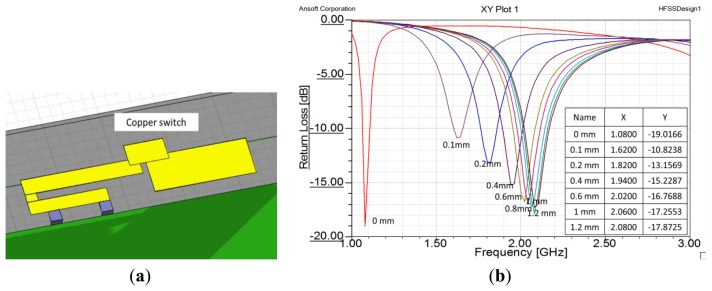
(**a**) Design model of copper sheet switch; (**b**) Result of height variation of switch. The horizontal axis represents frequency and the vertical axis represents return loss.

Next, we created a small piece of copper to serve as a switch. The piece of copper was 7 mm × 4 mm. Figure 8a shows the copper sheet set above the middle of the gap. If the copper sheet adhered to the ends of the two antenna strips, the strips were connected together and the effective length of the antenna was increased. If the copper sheet was separated from the two antenna strips, they were disconnected and the effective length of the antenna was decreased. The results of height variation of the switch are shown in Figure 8b. That figure shows that if the copper sheet left the plane of the antenna, the operating the frequency increased radically to 2.1 GHz. From the above results, we believe that using an IPMC actuator to control the height of the copper sheet is a proper method to achieve a dual-band inverted-F antenna.

### 3.3. Fabrication and Measurement of IPMC inside a Mobile Device

**Figure 9.**
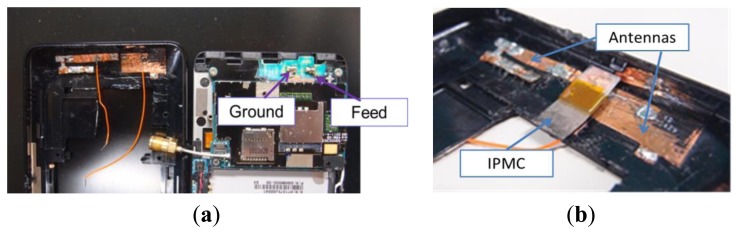
(**a**) Structure of antennas in mobile phone model; (**b**) IPMC switch connects two antenna strips.

After simulation, we fabricated a similar antenna in a mobile phone. We pasted two strips of copper tape as an inverted-F antenna and extended the path on the shell, as shown in Figure 9a. Semi-rigid coaxial cable with a Sub Miniature version A (SMA) connector was used. When the back cover and the main body of the cell phone were combined, the antenna could touch the two metal contact points, one of which served as the grounded point and the other as the feed point, which was connected to the semi rigid coaxial cable with the SMA connector. We used the IPMC actuator to connect the antennas and extend the path, as shown in Figure 9b.

**Figure 10.**
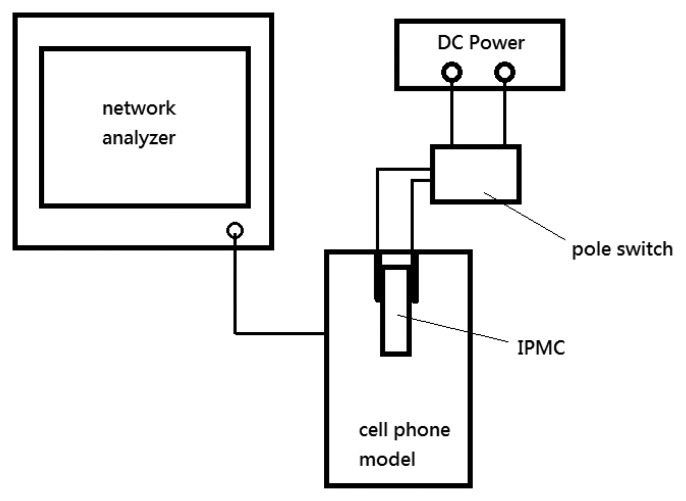
Schematic diagram of operation frequency and return loss measured by network analyzer.

We measured the operating frequency *versus* return loss with a model number E5061B network analyzer (Agilent, Santa Clara, CA, USA). The network analyzer was set to output impulse signals at a range of frequencies while measuring the reflected amplitude at the same time. Thus, the resonant frequency of the antenna could be determined. As illustrated in Figure 10, we used a switch to open or close the voltage applied on the IPMC actuator. This device was connected to the Agilent network analyzer to measure the operation frequency and the return loss. The DC power supply was used to control the IPMC switch.

**Figure 11.**
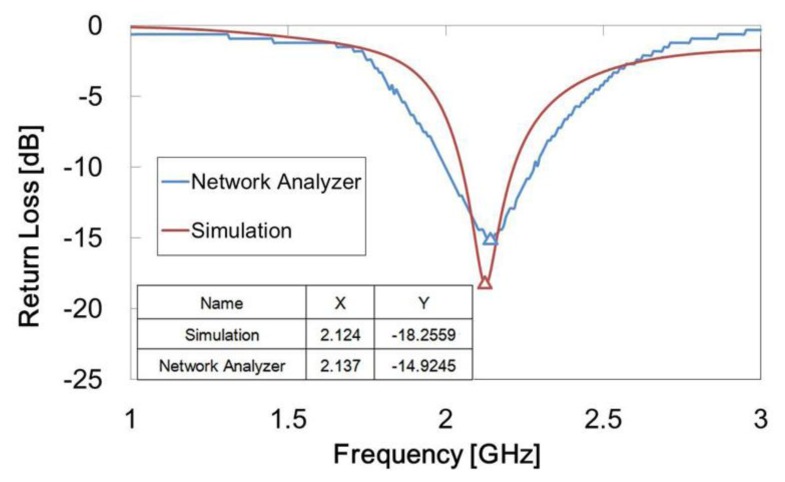
Line chart of experimental results compared with simulation when IPMC switch operates. The horizontal axis represents frequency and the vertical axis represents return loss.

The inverted-F antenna we designed was measured to have a best response of −14.92 dB at 2.14 GHz. In comparison, the previous simulation result was −18.26 dB at 2.12 GHz. A line chart of the experimental results and simulation are shown in Figure 11. The operating frequency peak values of both the experiment and the simulation were near 2.1 GHz, but the return loss peak value of the experiment was slightly higher than that of the simulation. Also, the bandwidth of the experiment was slightly wider than that of the simulation. This could be due to the antenna fabrication tolerance.

**Figure 12.**
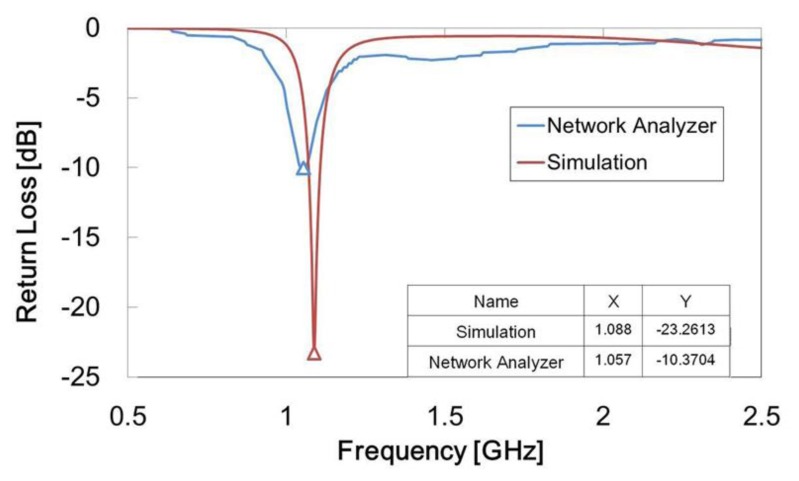
Line chart of experimental results compared with simulation when IPMC switch is off. The horizontal axis represents frequency and the vertical axis represents return loss.

Figure 12 shows the results of extending the antenna path when the IPMC switch contacted the inner ends of the two antenna strips. The operating frequency shifted to 1.06 GHz and the return loss was −10.37 dB, which means our design was able to attain the goal of changing the operating frequency to 1.1 GHz. The simulation result was −13.26 dB at 1.09 GHz. Although the return loss was higher, it was still within an acceptable range, below −10 dB. When the voltage was switched back to 3 V, the condition returned to the off state and the operating frequency returned to 2.1 GHz. Table 1 compares the measurement results. The operating frequency clearly changed between the two switch states, and both return losses were less than −10 dB. We operated this IPMC switch several times, and the response time results are presented in Table 2. The average response time from the off state to the on state (from 2.1 to 1.1 GHz) was 1.14 s, and the response time from the on state to the off state (from 1.1 to 2.1 GHz) was about 1.34 s. These response times demonstrate that the operating frequency could be changed easily and fast. We feel confident in stating that the effective length of the antenna was successfully changed by the IPMC switch.

## 4. Reliability

As previously mentioned, the IPMC bends because of the hydrated positive ions carrying water that moves toward the anode. If the IPMC is exposed to air without the water supply, the water can evaporate and the water uptake can be decreased. Furthermore, we usually operated the IPMC actuator at 3 V, at which electrolysis of the water is inevitable. These defects degrade the bending performance and increase the difficulty of designing the mechanism.

To solve the above-mentioned questions, we used propylene carbonate electrolyte with lithium perchlorate (LiClO_4_) for the IPMCs. The electrolyte uses propylene carbonate as its solvent and Li cations as its free ions. Propylene carbonate is a polar solvent that has very low vapor pressure and does not evaporate easily at room temperature. It also has a high dielectric permittivity that allows larger IPMC voltages without the problem of electrolysis.

In this work, we prepared propylene carbonate (99.5% w/w) 25 mL as solvent and added lithium perchlorate (LiClO_4_) 0.6 M. The IPMCs were immersed into the propylene carbonate solution for 3 days. The IPMCs were 10 mm × 6 mm (sample A) and 10 mm × 5 mm (sample B), and we measured the tip displacement and weight of the IPMCs operated at 3.5 Volts DC each day. After each daily measurement, we stored the IPMC samples at room temperature.

**Figure 13.**
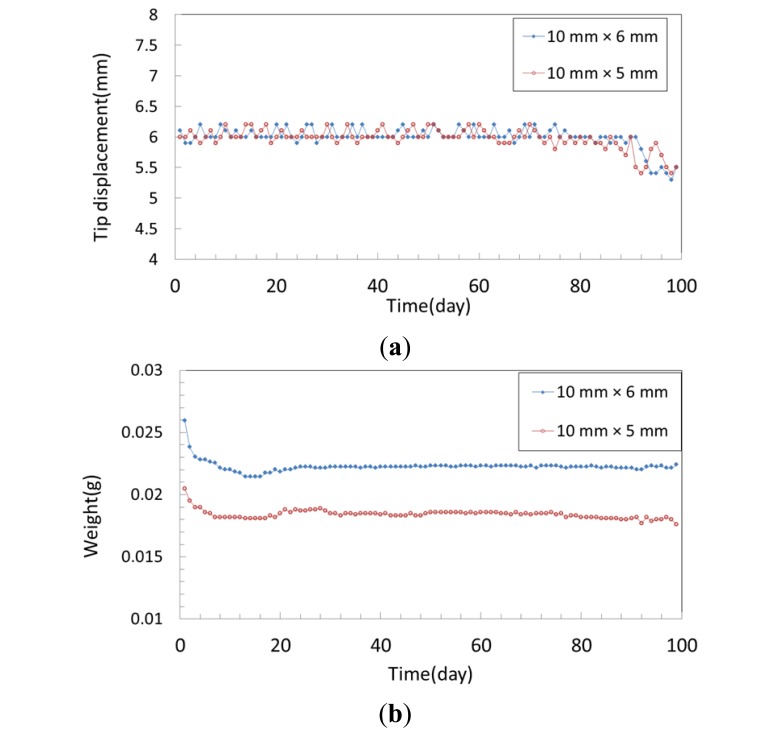
(**a**) Variation in tip displacement of IPMC with propylene carbonate solution. (**b**) Variation in weight of IPMC with propylene carbonate solution.

Figure 13a is a statistical chart of variation in tip displacement of the IPMC after it was soaked in propylene carbonate. The tip displacements of both sample A (10 mm × 6 mm) and sample B (10 mm × 5 mm) were about 6 mm over three months. There was a slight drop after day 90. These results show that the performance of the IPMCs with propylene carbonate was steady, without evaporation or electrolysis. Figure 13b shows the variation in weight of the IPMC after being soaked in propylene carbonate. The weights of sample A and sample B were about 0.026 g and 0.02 g on the first day, respectively. After being actuated several times for several days, their weights fell to 0.022 g and 0.018 g on day 8, respectively. Thereafter, the weights remained stable. We think that the weight loss in the beginning was due to evaporation of the residual water content or gradual electrolysis under the applied voltage. After that, only propylene carbonate remained.

**Figure 14.**
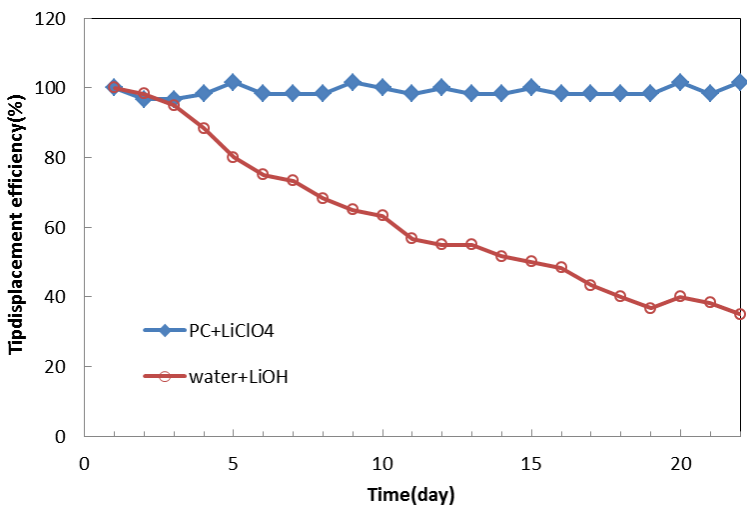
Variation of normalized tip displacement of IPMC with propylene carbonate and water solution.

We normalized the data of the tip displacement of the IPMC with propylene carbonate solution and compared it to that with lithium hydroxide water solution, as shown in Figure 14. As time went by, the tip displacement of the IPMC with propylene carbonate solution remained almost the same. In contrast, with water solution, it dropped to less than 40% after day 20. In the future, attempts should be made to control the deposition density of the metal electrodes to minimize solvent loss. Coating other metals on the surface of the IPMC to make the electrodes denser could reduce the surface resistance and evaporation. Another way to eliminate solvent loss would be to encapsulate the IPMC. We feel confident that the lifetime of the IPMC in air can be effectively prolonged. From these results, we believe that propylene carbonate is a promising replacement for the water solution and is possible to make fast-speed polymer actuators [13] in future.

## 5. Conclusions

In this paper, we have demonstrated a new RF switch that is light in weight and has a low driving power, large deformation, and frequency-shifting capability. After our experiments, we investigated a setup with a bridge style switch. This switch used the IPMC as an actuator to move a copper sheet upward and downward. When the IPMC-bridge was turned off, the antenna could be considered to be longer due to the copper sheet connecting the antennas and thereby extending the path. Simulation results showed that the operating frequency changed from 1.09 to 2.12 GHz and that the return losses at both frequencies were both less than −10 dB. Using a network analysis system, we determined that the original operating frequency of the antenna, 1.07 GHz, could be changed to 2.14 GHz when the IPMC switch was on. It has been demonstrated experimentally that the operating frequency can be effectively changed from a low band to a high band.

To prolong the lifetime of the IPMC in air, we used propylene carbonate electrolyte with LiClO_4_. The IPMCs were still functional over three months after being actuated daily at 3.5 Volts DC voltage. Our device has lower driving voltage, which is suitable for application in cell phones. The IPMC switch is a potential solution to integrating antenna systems on mobile devices.

## Figures and Tables

**Table 1.** Result of operating frequency and return loss of reconfigurable antenna in mobile device.

**Voltage(V)**	**Switch state**	**Operating frequency(GHz)**	**Return loss(dB)**
0	on	1.06	−10.4
3	off	2.13	−14.9

**Table 2.** Response time with changing frequency in a reconfigurable antenna.

**Switch Status**	**1st (s)**	**2nd (s)**	**3rd (s)**	**4th (s)**	**Average (s)**
**Off to On**	1.12	1.24	1.19	1.01	1.14
**On to Off**	1.37	1.52	1.19	1.29	1.3425
